# Negative SARS-COV-2 pleural effusion in breast carcinoma coincide with COVID-19 infection: Case report

**DOI:** 10.1016/j.amsu.2021.102283

**Published:** 2021-04-18

**Authors:** Eko Setiawan, Dian Ayu Listiarini, Shafira Zahra Ovaditya

**Affiliations:** a Department of Surgery, Medical Faculty, Sultan Agung Islamic University / Sultan Agung Islamic Hospital, Semarang, 50164, Indonesia; b Department of Anesthesiology, Medical Faculty, Sultan Agung Islamic University / Sultan Agung Islamic Hospital, Semarang, 50164, Indonesia

**Keywords:** Case report, COVID-19, Pleural effusion, Breast carcinoma, SARS-CoV-2

## Abstract

**Introduction:**

and Importance: COVID-19 infection presents various symptoms that may resemble signs and symptoms of other underlying diseases. Pleural effusion in a confirmed COVID-19 patient with a history of malignancy is found to be rare, and the exact pathogenesis is still unclear. Hence RT-PCR COVID-19 assay from pleural effusion fluid is essential.

**Case presentation:**

A 62-year-old female patient was admitted to the emergency department with a complaint of shortness of breath and headache. Previously, the patient was diagnosed with stage III breast carcinoma. The chest radiograph showed massive pleural effusion. The SARS-CoV-2 was found in the nasopharyngeal and oropharyngeal sample, but the RT-PCR COVID-19 assay of pleural fluid was negative.

**Clinical discussion:**

Pleural effusion can be an uncommon manifestation of COVID-19, but there are many other etiologies. Malignancy is a commonly encountered underlying cause of the pleural effusion. Since it presents similar respiratory signs and symptoms, awareness of possible etiologies is pivotal. A strict examination, assessment, and protocol should be done to prevent the intervention's potential hazard.

**Conclusion:**

Pleural effusion related to COVID-19 infection can resemble the clinical presentation in a patient with a malignancy history. SARS-CoV-2 can be found in the nasopharyngeal and oropharyngeal sample but absent in pleural effusion fluid.

## Highlights

• Pleural effusion related to COVID-19 infection can resemble the clinical presentation in a patient with a malignancy history.
• SARS-CoV-2 can be found in the nasopharyngeal and oropharyngeal sample but absent in pleural effusion fluid.
• The SARS-CoV-2 can be absent from pleural effusion fluid in a confirmed COVID-19 patient.
• Prompt screening and RT-PCR COVID-19 assay from pleural effusion fluid are essential.

## 1 Background

**Fig. 1:**
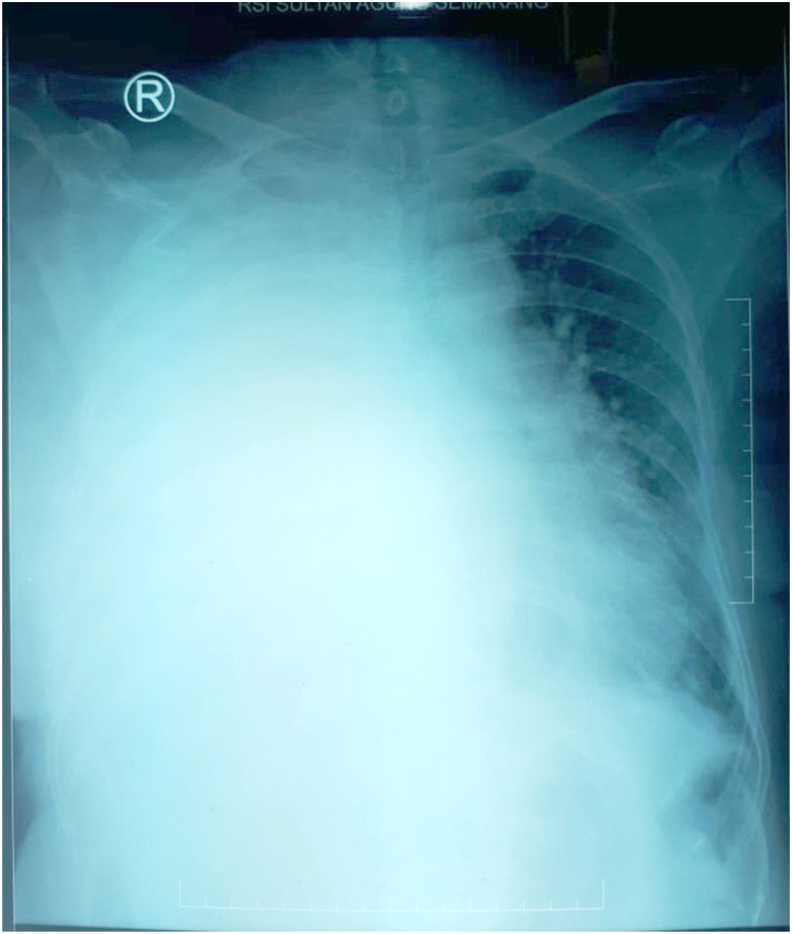
Initial Chest X-Ray showed massive pleural effusion on the right pleural cavity.

**Fig. 2:**
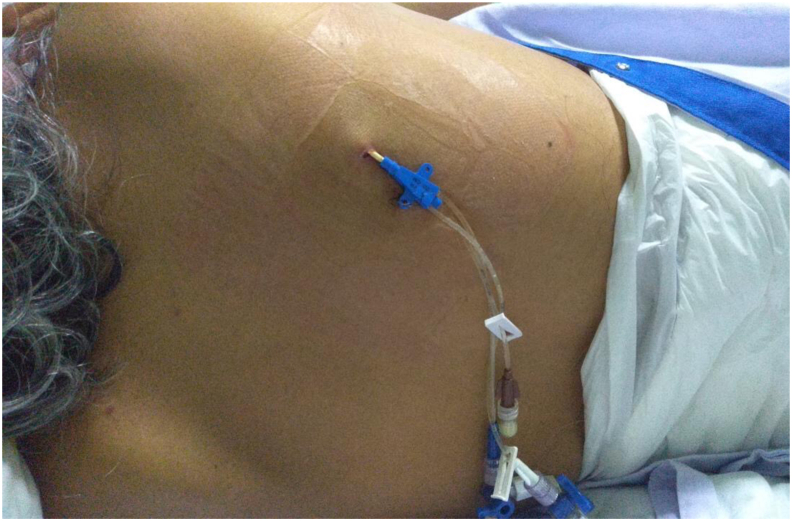
Modified pigtail using seven french central venous catheters inserted with the posterior approach.

**Fig. 3:**
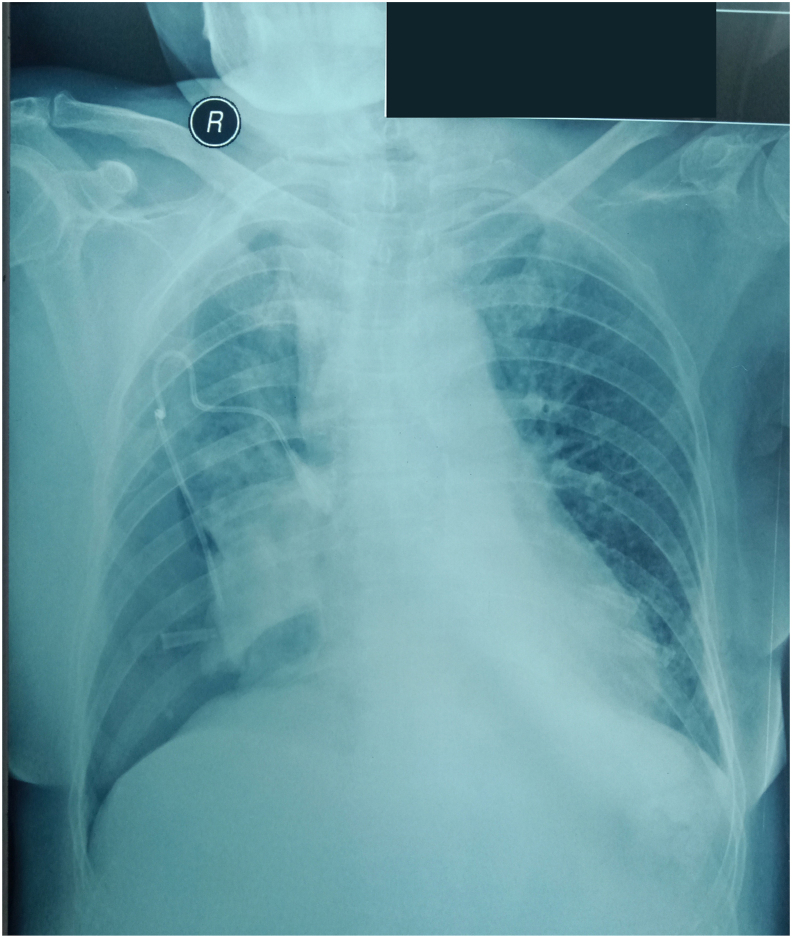
Chest X-Ray after the 11th day of pleural fluid drainage showed no effusion.

COVID-19 is manifested with a variety of symptoms that may resemble other underlying diseases. Pleural effusion in a confirmed COVID-19 patient with a history of malignancy is found to be rare, and the exact pathogenesis is still unclear. A strict protocol and assessment should be done before invasive intervention to prevent the potential hazard [1]. Detection of SARS-CoV-2 in pleural fluid had been reported in the previous study [2]. Meanwhile, in this case, SARS-CoV-2 was found in the nasopharyngeal and oropharyngeal specimen but absent in pleural effusion fluid. This case has adjusted to the SCARE criteria [3]. (see Fig. 1, Fig. 2, Fig. 3)

## 2 Presentation of case

A 62-year-old female patient was admitted to the emergency department with a complaint of shortness of breath and headache of 7 days. On examination, the patient's blood pressure was 200/108 mmHg, heart rate was 127/min, respiration rate was 28/min, the temperature was 37,1°celcius, and oxygen saturation was 99% on room air. The patient's appearance was tachypneic. Previously, the patient was diagnosed with stage III breast carcinoma for seven months, had already done a mastectomy, and completed six cycles of adjuvant chemotherapy before her presentation. The chest examination showed a decrease in lung sound in auscultation and dullness in percussion. The patient's laboratory tests showed quantitative C-reactive protein 9,1 mg/L, Absolute lymphocyte count 1,11 × 10^3^/μL, Neutrophil-to-lymphocyte ratio 6,2, and D-dimer 1,67 mg/L.

The chest radiograph revealed massive pleural effusion in the right pleural cavity. CT scan cannot be performed due to limited resources in our hospital. A Real-Time Polymerase Chain Reaction (RT-PCR) COVID-19 assay from oropharyngeal and nasopharyngeal swab was found to be positive. A modified pigtail device using 3-lumen 7 french central venous catheter was inserted into the right pleural cavity by a surgeon, draining yellowish serous fluid. A pleural fluid sample was sent for RT-PCR COVID-19 assay, culture and sensitivity test, gram staining, and cytology. RT-PCR COVID-19 from the pleural fluid analysis showed a negative result. The culture test and gram staining test were negative for bacteria, fungi, and leukocytes. The malignant cell was negative in cytological analysis.

On the 7th day of hospitalization, there was no pleural fluid production. The patient's clinical presentation improved, no recurrence during the hospital course, and the patient was finally discharged from the 13th day.

## 3 Discussion

COVID-19 is a disease caused by SARS-CoV-2 presenting in various symptoms that may resemble signs and symptoms of other underlying disease [4]. Pleural effusion can present as an uncommon manifestation of COVID-19, but there are many other etiologies [5]. Malignancy is a commonly encountered underlying cause of pleural effusion [6]. Since the clinical presentation shows similar respiratory signs and symptoms, awareness of possible etiologies is pivotal.

In this case, the patient was previously diagnosed with stage III breast carcinoma and admitted to the emergency department due to shortness of breath caused by massive pleural effusion. The patient underwent pleural drainage as a therapeutic and diagnostic tool to find the underlying cause of pleural effusion, whether it is caused by malignancy or as a manifestation of SARS-CoV-2 infection. So that, we performed RT-PCR COVID-19 assay, culture and sensitivity test, gram staining, and cytology from pleural fluid. The SARS-CoV-2 was found in the nasopharyngeal and oropharyngeal sample, but the RT-PCR COVID-19 assay of pleural fluid was negative. Although the absence of SARS-CoV-2 in the pleural fluid provides a reassurance, it gives a current concern about the potential hazard of pleural effusion drainage. A strict examination, assessment, and protocol should be done in all patients with COVID-19 mimicking presentation.

The exact mechanism of pleural effusion related to COVID-19 is still uncertain, but it provides an adverse prognostic sign indicating super-infection of bacteria in COVID-19 pneumonia [7]. Despite this case, the culture and gram staining result showed negative results, suggesting no super-infection. The presence of pleural effusion may hinder lung expansion, inadequate ventilation, worsen clinical outcomes, and lead to mortality [8]. Fortunately, the patient presented in this case had clinical improvement on the 7th day, after all of the pleural fluid had been successfully removed. Successful pleural fluid removal causes the lung to be better expanded, which contributes to better ventilation [2].

Although our findings showed no direct involvement of pleural fluid, further study regarding the importance of pleural effusion fluid analysis from the COVID-19 patient is needed to identify the presence of SARS-CoV-2 in pleural effusion fluid.

## 4 Conclusion

Pleural effusion related to COVID-19 infection can resemble the clinical presentation in a patient with a malignancy history. SARS-CoV-2 can be found in the nasopharyngeal and oropharyngeal sample but absent in pleural effusion fluid.

## Ethics approval

Ethical approval is not required at our Institution for case reports.

## Sources of funding

We report no involvement of any sponsor or funding body for this study.

## Authors’ contributions

ES conceptualized the first draft and finalized the manuscript. DAL supplied the imaging data and critically revised imaging technique. SZO wrote the manuscript. All authors read and approved the final manuscript.

## Consent for publication

Written informed consent was obtained from the patient for publication of this case report and accompanying images. A copy of the written consent is available for review by the Editor-in-Chief of this journal on request.

## Trial registry number

Not applicable for case reports.

## Guarantor

Eko Setiawan.

## Provenance and peer review

Not commissioned, externally peer-reviewed.

## Declaration of competing interest

All authors have declared that they have no potential competing interests.
